# Simulation extractable versions of Groth’s zk-SNARK revisited

**DOI:** 10.1007/s10207-023-00750-7

**Published:** 2023-09-05

**Authors:** Oussama Amine, Karim Baghery, Zaira Pindado, Carla Ràfols

**Affiliations:** 1 University of Oslo, Oslo, Norway; 2 COSIC, KU Leuven, Leuven, Belgium; 3 Dusk, Amsterdam, Netherlands; 4 Universitat Pompeu Fabra, Barcelona, Spain

**Keywords:** NIZK, Zk-SNARK, Strong simulation extractability, Algebraic group model, Random oracle model

## Abstract

Zero-knowledge succinct non-interactive arguments of knowledge (zk-SNARKs) are the most efficient proof systems in terms of proof size and verification. Currently, Groth’s scheme from EUROCRYPT 2016, $\textsf{Groth16}$, is the state-of-the-art and is widely deployed in practice. $\textsf{Groth16}$ is originally proven to achieve knowledge soundness, which does not guarantee the non-malleability of proofs. There has been considerable progress in presenting new zk-SNARKs or modifying $\textsf{Groth16}$ to efficiently achieve *strong* Simulation extractability, which is shown to be a necessary requirement in some applications. In this paper, we revise the Random oracle based variant of $\textsf{Groth16}$ proposed by Bowe and Gabizon, BG18, the most efficient one in terms of prover efficiency and CRS size among the candidates, and present a more efficient variant that saves 2 pairings in the verification and 1 group element in the proof. This supersedes our preliminary construction, presented in CANS 2020 (Baghery et al. in CANS 20, volume 12579 of LNCS, Springer, Heidelberg. pp 453-461, 2020), which saved 1 pairing in the verification, and was proven in the generic group model. Our new construction also improves on BG18 in that our proofs are in the algebraic group model with Random Oracles and reduces security to standard computational assumptions in bilinear groups (as opposed to using the full power of the generic group model (GGM)). We implement our proposed simulation extractable zk-SNARK (SE zk-SNARK) along with BG18 in the Arkworks library, and compare the efficiency of our scheme with some related works. Our empirical experiences confirm that our SE zk-SNARK is more efficient than all previous simulation extractable (SE) schemes in most dimensions and it has very close efficiency to the original $\textsf{Groth16}$.

## Introduction

Non-interactive zero-knowledge (NIZK) proof systems [11] are a fundamental family of cryptographic primitives that have appeared recently in a wide range of practical applications. A NIZK proof system allows a party to prove that for a public statement $\vec {x}$, she knows a witness $\vec {w}$ such that $(\vec {x}, \vec {w}) \in \textbf{R}$, for some relation $\textbf{R}$, without leaking any information about $\vec {w}$ and without interaction with the verifier. Due to their impressive advantages, NIZK proof systems are used ubiquitously to build larger cryptographic protocols and systems.

Zero-knowledge Succinct Arguments of Knowledge (zk-SNARKs) are among the most interesting NIZK proof systems in practice, as they allow to generate very short proofs for NP complete languages, which can be verified in less than 10 milliseconds [20, 22]. Zk-SNARKs have had a tremendous impact in practice and they have found numerous applications, including verifiable computation systems [32], privacy-preserving (PP) cryptocurrencies [8], PP smart contract systems [28], PP proof-of-stake protocols [24], and efficient ledger verification protocols [13], are some of the best known applications that use zk-SNARKs to prove different statements very efficiently while guaranteeing the privacy of the prover. Because of their practical importance, particularly in large-scale applications like blockchains, even minimal savings especially in proof size or verification cost are considered to be relevant.

In 2016, Groth [22] introduced the most efficient zk-SNARK for Quadratic Arithmetic Programs or QAPs, which is still the state-of-the-art construction, $\textsf{Groth16}$. It is constructed using bilinear groups and its proof is 3 group elements (2 from $\mathbb {G}_1$ and 1 from $\mathbb {G}_2$) and the cost of verification is dominated by 3 pairing computations. In the original paper, it is proven to achieve knowledge soundness in the generic group model (GGM). In 2018, Fuchsbauer, Kiltz, and Loss [19] defined the algebraic group model (AGM) and reproved its security in this weaker model. The proof of $\textsf{Groth16}$ is malleable, as it is shown in [23]. Generating non-malleable proofs is a necessary requirement in building various cryptographic schemes, including *universally composable* protocols [24, 28], cryptocurrencies (e.g. Zcash) [8], signature-of-knowledge schemes [23], etc. Practical systems like Zcash cryptocurrency [8] that uses the original $\textsf{Groth16}$ [22] make extra efforts to ensure the non-malleability of transactions and the proof of underlying proof system. Considering such concerns, in practice, it is important to have a stronger notion of knowledge soundness, known as (strong) Simulation Extractability (SE). This notion guarantees that a valid witness can be extracted from any adversary producing a proof accepted by the verifier, even after seeing an arbitrary number of simulated proofs.

There have been considerable efforts to construct new SE zk-SNARKs or refine Groth’s zk-SNARK to achieve SE and guarantee the non-malleability of proofs. Firstly, in 2017 Groth and Maller [23] proposed an SE zk-SNARK, which is very efficient in terms of proof size but very inefficient in terms of Common Reference String ($\textsf{crs}$) size and prover time. They also showed how one can use SE zk-SNARKs to build Signature of Knowledge (SoK) schemes [16] with *succinct* signatures. In 2018 Bowe and Gabizon [14] proposed a less efficient construction in terms of proof size (5 group elements vs 3 in the original version) based on $\textsf{Groth16}$ which needs a Random Oracle (RO) (apart from GGM) which returns group elements, but with almost no overhead in the $\textsf{crs}$ size or additional cost for the prover. In [29], Lipmaa proposed several constructions, including an efficient QAP-based SE zk-SNARK in terms of proof size and with the same verification complexity as [14, 23], but less efficient in terms of $\textsf{crs}$ size and prover time compared to [14] and $\textsf{Groth16}$. In [2], Atapoor and Baghery used the traditional OR technique to achieve SE in $\textsf{Groth16}$. Their variant requires 1 pairing less for verification in comparison with previous SE constructions, however it comes with an overhead in proof generation, $\textsf{crs}$ size, and even larger overhead in the proof size. For a particular instantiation they add $\approx 52.000$ constrains to the underlying QAP instance, which adds fixed overhead to the prover and $\textsf{crs}$ size, that can be considerable for mid-size circuits. They show that for a circuit with $10 \times 10^6$ Multiplication (Mul) gates, their prover is about $10\%$ slower, but it can be slower for circuits with less than $10 \times 10^6$ gates. In [26], Kim, Lee, and Oh proposed a QAP-based SE zk-SNARK with the same $\textsf{crs}$ size and prover time compared to [29], but with slightly shorter proofs and more efficient verification.

These works also differ significantly in the assumptions they make for security. The scheme of Groth and Maller [23] is based on a knowledge assumption and other falsifiable computational assumptions, and they are all q-type assumptions where *q* is the size of the circuit. In this work, the authors avoid the generic group model by making a concrete knowledge assumption that is essential for extracting the witness. On the other hand, the work of Bowe and Gabizon [14] uses the full power of the generic group model to prove the security. The construction of Bowe and Gabizon uses the generic group model plus the assumption that a certain hash function to group elements is a random oracle. All the constructions of Lipmaa [29] are proven secure in a weaker notion of the AGM, where the adversary has access to a random oracle that allows it to sample random elements obliviously in the group, i.e. without knowing the random oracles.

Recently, Baghery, Kohlweiss, Siim, and Volkhov [6] explored another direction. Instead of modifying $\textsf{Groth16}$ to achieve *strong* SE, they first show that the original construction of $\textsf{Groth16}$ achieves *weak* SE with non-black-box extraction. Weak SE allows proof randomization, therefore the proof is malleable, while it guarantees that a proof cannot be changed to prove a new statement. Then, considering the first result, they proposed two efficient constructions of $\textsf{Groth16}$ that achieve weak SE with *black-box* extraction which is shown to be necessary for UC-security. Both *weak* and *strong* SE zk-SNARKs can be lifted to achieve black-box simulation extractability with a simpler compiler [3, 6], rather than with the COCO framework [27] which is constructed to lift (knowledge) sound NIZK proofs systems to achieve black-box SE. However, to realize the standard ideal functionality defined for NIZK arguments, one would need to use a strong SE NIZK with black-box extraction [21]. Therefore, constructing a more efficient strong SE zk-SNARK, would also allow to build more efficient *black-box* SE zk-SNARK to be used in UC-secure protocols.

**Table 1:** A comparison of our proposed variations of $\textsf{Groth16}$ along with the other SE zk-SNARKs for arithmetic circuit satisfiability with *n* Mul gates (constraints) and *m* wires (variables), of which *l* are public input wires (variables)

SNARK	SE	Model	CRS size	Prover	Proof	Verifier	VE
Groth [6, 19, 22]	WSE	AGM	$m + 2n - l$ $\mathbb {G}_1$	$m + 3n - l$ $E_1$	2 $\mathbb {G}_1$	*l* $E_1$	1
			*n* $\mathbb {G}_2$	*n* $E_2$	1 $\mathbb {G}_2$	3 P	
Groth-Maller [23]	SSE	GGM	$2m + 4n$ $\mathbb {G}_1$	$2m + 4n - l$ $E_1$	2 $\mathbb {G}_1$	*l* $E_1$	2
			2*n* $\mathbb {G}_2$	2*n* $E_2$	1 $\mathbb {G}_2$	5 P	
Bowe-Gabizon [14]	SSE	GGM	$m + 2n - l$ $\mathbb {G}_1$	$m + 3n - l$ $E_1$	3 $\mathbb {G}_1$	*l* $E_1$	2
		ROM	*n* $\mathbb {G}_2$	*n* $E_2$	2 $\mathbb {G}_2$	5 P	
Atapoor-Baghery [2]	SSE	GGM	$m' + 2n' - l$ $\mathbb {G}_1$	$m' + 3n' - l$ $E_1$	4 $\mathbb {G}_1$	$l'+2$ $E_1$	2
			$n'$ $\mathbb {G}_2$	$n'$ $E_2$	2 $\mathbb {G}_2$	4 P	
					+ 2 $\lambda$		
Lipmaa [29]	SSE	AGM	$m + 3n - l$ $\mathbb {G}_1$	$m + 4n - l$ $E_1$	3 $\mathbb {G}_1$	$l+1$ $E_1$	2
		tag-based	*n* $\mathbb {G}_2$	*n* $E_2$	1 $\mathbb {G}_2$	5 P	
KLO [26]	SSE	HAK	$m + 3n - l$ $\mathbb {G}_1$	$m + 4n - l$ $E_1$	2 $\mathbb {G}_1$	$l+1$ $E_1$, 1 $E_2$	1
		LCR	*n* $\mathbb {G}_2$	*n* $E_2$	1 $\mathbb {G}_2$	3 P	
BPR [7], Appendix A	SSE	GGM	$m + 2n - l$ $\mathbb {G}_1$	$m + 3n - l$ $E_1$	3 $\mathbb {G}_1$	*l* $E_1$, 1 $E_2$	2
		CRH	*n* $\mathbb {G}_2$	*n* $E_2$	2 $\mathbb {G}_2$	1 $E_T$, 4 P	
Sect. 3	SSE	AGM	$m + 2n - l$ $\mathbb {G}_1$	$m + 3n - l$ $E_1$	2 $\mathbb {G}_1$	*l* $E_1$, 1 $E_2$	1
		ROM	*n* $\mathbb {G}_2$	*n* $E_2$	2 $\mathbb {G}_2$	3 P	

A typical set of values is $n=m=10^6$ and $l=10$. In the case of $\textsf{crs}$ size and prover’s computation we omit constants. In [23], *n* Mul gates and *m* wires translate to 2*n* squaring gates and 2*m* wires. In [2], SE is achieved with an OR approach which requires to add constraints and variables, resulting in $n' \approx n + 52.000$, $m' \approx m + 52.000$, and $l' = l + 4$. $\mathbb {G}_1, \mathbb {G}_2$ and $\mathbb {G}_T$: group elements, $E_i$: exponentiation in group $\mathbb {G}_i$, $M_i$: multiplication in group $\mathbb {G}_i$, *P*: pairings

*GGM* Generic group model, *ROM* Random oracle model, *AGM* Algebraic group model, *HAK* Hash algebraic knowledge assumption, *LCR* Linear collision resistance hash functions, *CRH* Collision resistant hash, *VE* Number of verification equations, *WSE* Weak simulation extractable, *SSE* Strong simulation extractable

***Our Contributions***. Our main contribution is to revise the simulation extractable variants of $\textsf{Groth16}$, presented in [14] and [2], to achieve a better efficiency and get the best of both constructions. Namely, achieving *strong* simulation extractability in $\textsf{Groth16}$ with minimal overhead.

Our focus is mainly on Bowe and Gabizon’s variation [14] which has the most efficient prover and the shortest $\textsf{crs}$ among other (strong) SE zk-SNARKs [2, 14, 23, 26, 29], while it uses a RO which returns group elements. To achieve (strong) simulation extractability, their prover replaces all the original computations which depend on some parameter $\delta$ given in the $\textsf{crs}$ by some $\delta '$ and the prover must give $[\delta ']_2$ and a proof of knowledge (PoK) of the DLOG of $[\delta ']_2$ w.r.t $[\delta ]_2$. Using this technique, they present a variation that has the same CRS as $\textsf{Groth16}$, almost the same prover as $\textsf{Groth16}$, 2 new elements in the proof (one from $\mathbb {G}_1$ and the other from $\mathbb {G}_2$), and an additional verification equation that adds 2 pairing operations to the verification of $\textsf{Groth16}$.

In this paper, using the same approach [14] and some subtle modifications, we construct a *strong* SE zk-SNARK that results in the most efficient (strong) simulation extractable variant of $\textsf{Groth16}$ in terms of $\textsf{crs}$ size, prover complexity, and verification time. Our SE zk-SNARK uses some sophisticated modification of Boneh-Boyen signatures [12] to prove knowledge of the DLOG of $\delta '$ which requires 1 less $\mathbb {G}_1$ element in the proof, and 2 pairings less in the verification in comparison with the argument of Bowe and Gabizon [14], but at the cost of one additional exponentiation in the verification. Our construction supersedes and improves a preliminary version of this work presented at CANS 2020 [7], where in all constructions verification required at least one additional pairing and proofs were in the GGM.

Our construction modifies the proof generation of $\textsf{Groth16}$ slightly and include the PoK of the DLOG of $[\delta ']_2$ w.r.t $[\delta ]_2$ inside the original proof of $\textsf{Groth16}$. Using this, we manage to save 1 element in the proof, and 2 pairings in the verification of Bowe and Gabizon’s construction [14], at the cost of a single exponentiation in $\mathbb {G}_2$ in the verification. This construction shows that using a random oracle, we can achieve strong SE in $\textsf{Groth16}$, at the cost of one additional $\mathbb {G}_2$ element in the proof, and one new exponentiation in $\mathbb {G}_2$ in the verification. In the case of verifying a larger number of proofs where verifiers of our constructions gain efficiency by using *multi-scalar exponentiations*, our construction achieves almost the same efficiency as $\textsf{Groth16}$.

Table 1 presents a comparison of our proposed variant of $\textsf{Groth16}$ with several other constructions for a particular instance of arithmetic circuit satisfiability. As it can be seen, in comparison with Bowe and Gabizon’s construction [14], our construction retains most of the properties requires 2 less pairing in the verification, at the cost of 1 additional exponentiation in the verification. We also compare our construction with the results initially obtained and presented in CANS 2020 [7]. We note that in both our constructions, the hash function maps into $\mathbb {Z}_p$ and not to a source group as in [14], which is an additional practical advantage. In comparison with Atapoor and Baghery’s construction [2], both of our variants have a negligible overhead in the proof generation and $\textsf{crs}$ size, and a smaller overhead in proof size. Above all, our best construction, requires 3 parings in the verification, instead of 4.^1^ We reduce security to a q-DLOG in the AGM with random oracles, where *q* is the size of the circuit. In contrast, our preliminary result [7] was in the GGM but only required the hash function to be collision resistant.

As a part of our contribution, we also present an open-source prototype implementation of our presented constructions and Bowe and Gabizon’s scheme in the Arkworks library, which currently is one of the most popular ecosystems written in Rust for developing and programming with zk-SNARKs. Then, we use our implementations along with the implementations of $\textsf{Groth16}$ [22] and Groth-Maller [23], which already exist in $\textsf{Arkworks}$ library, and present a comprehensive benchmark for the relevant simulation extractable zk-SNARKs [14, 22, 23]. Full details of our empirical analysis are reported in Sect. 4, in Table 2. As we expected, the implementation results show that, our new construction is more efficient than the first one, and also it is more efficient than all previous SE zk-SNARKs in most dimensions and more importantly it has a very close efficiency profile to the original $\textsf{Groth16}$, particularly when we need to verify a large number of proofs.

Finally, we highlight that using the technique proposed in [23], both of or proposed SE zk-SNARKs can be turned into *succinct* SoK schemes, which would be more efficient than previous constructions. In general, due to relying on non-falsifiable assumptions, succinct SoK schemes have better efficiency in comparison with constructions that are built under standard assumptions [5, 10, 16]. We also note that to achieve strong (non-black-box) SE, our proposed zk-SNARKs require minimal changes in comparison with the original $\textsf{Groth16}$. Therefore, one can use the same compiler or ad-hoc approach proposed in [3] and [6], respectively, to construct a more efficient strong *black-box* SE zk-SNARK for UC-protocols [21].

*Organization.* In Sect. 2, we introduce notation, the relevant security definitions, and recall the Boneh-Boyen signature scheme. In Sect. 3, we present our new and the most efficient SE zk-SNARK, that has very close efficiency to the $\textsf{Groth16}$. We evaluate the practical efficiency of both presented constructions in Sect. 4 using a prototype Rust implementation in $\textsf{Arkworks}$ library. We also compare the efficiency of our constructions with several relevant SE zk-SNARKs in the same section. Finally we conclude the paper in Sect. 5. For the sake of completeness, in Appendix A, we also recall our first SE zk-SNARK [7] that relaxes the RO in Bowe and Gabizon’s scheme [14] to a collision resistant hash function, and also saves 1 pairing in the verification. We implement that scheme as well and include it in our benchmarks.

*Novelty.* Compared to the conference version published in CANS 2020 [7], this version includes a more efficient construction presented in Sect. 3, a prototype Rust implementation of our presented constructions along with Bowe and Gabizon’s scheme [14] in $\textsf{Arkworks}$ library, followed by a comprehensive efficiency comparison of relevant SE zk-SNARKs that are reported with details in Sect. 4.

## Preliminaries

### Notation and bilinear groups

We let $\textsf{BGgen}$ be a probabilistic polynomial time algorithm which on input $1^{\lambda }$, where $\lambda$ is the security parameter, returns the description of an asymmetric bilinear group $\textsf{gk}=(p,\mathbb {G}_1,\mathbb {G}_2,\mathbb {G}_T,e,\mathscr {P}_1,\mathscr {P}_2)$, where $\mathbb {G}_1,\mathbb {G}_2$ and $\mathbb {G}_T$ are groups of prime order *p*, the elements $\mathscr {P}_1, \mathscr {P}_2$ are generators of $\mathbb {G}_1,\mathbb {G}_2$ respectively, $e:\mathbb {G}_1\times \mathbb {G}_2\rightarrow \mathbb {G}_T$ is an efficiently computable, non-degenerate bilinear map, and there is no efficiently computable isomorphism between $\mathbb {G}_1$ and $\mathbb {G}_2$.

Elements in $\mathbb {G}_i$, are denoted implicitly as $[a]_i=a \mathscr {P}_i$, where $i \in \{1,2,T\}$ and $\mathscr {P}_T=e(\mathscr {P}_1,\mathscr {P}_2)$. With this notation, $e([a]_1,[b]_2) = [a]_1 [b]_2 = [ab]_T$. We extend this notation naturally to vectors and matrices. We denote by $\textsf{negl}(\lambda )$ an arbitrary negligible function in $\lambda$.

### Definitions

For an algorithm $\mathscr {A}$, let $\textbf{Im}(\mathscr {A})$ be the image of $\mathscr {A}$, i.e. the set of valid outputs of $\mathscr {A}$. By $y \leftarrow \mathscr {A}(x; r)$ we denote the fact that $\mathscr {A}$, given an input *x* and a randomizer *r*, outputs *y*.

We use the definitions of NIZK arguments from [22]. Let $\mathscr {R}$ be a relation generator, such that $\mathscr {R}(1^{\lambda })$ returns a polynomial-time decidable binary relation $\textbf{R}= \{(\vec {x}, \vec {w})\}$. Here, $\vec {x}$ is the statement and $\vec {w}$ is the witness. Security parameter $\lambda$ can be deduced from the description of $\textbf{R}$. The relation generator also outputs auxiliary information $\textsf{z}_{\textbf{R}}$ that will be given to the honest parties and the adversary. In our constructions, $\textsf{z}_{\textbf{R}}$ will be the description of a bilinear group. As in [22], $\textsf{z}_{\textbf{R}}$ is the value returned by $\textsf{BGgen}(1^{\lambda })$, and is given as an input to the parties.

Let $\mathsf {\mathscr {L}}_{\textbf{R}} = \{\vec {x}: \exists \vec {w}, (\vec {x}, \vec {w}) \in \textbf{R}\}$ be an NP-language. A *NIZK argument system* $\Psi$ for $\mathscr {R}$ consists of tuple of PPT algorithms $(\textsf{K}, \textsf{P}, \textsf{V}, \textsf{Sim})$, such that:

- CRS Generator: $\textsf{K}$ is a PPT algorithm that, given $(\textbf{R}, \textsf{z}_{\textbf{R}})$ where $(\textbf{R}, \textsf{z}_{\textbf{R}}) \in \textbf{Im}(\mathscr {R}(1^{\lambda }))$, outputs $\textsf{crs}:=(\textsf{crs}_{\textsf{P}}, \textsf{crs}_{\textsf{V}})$ and stores trapdoors of $\textsf{crs}$ as $\vec {\textsf{ts}}$. We distinguish $\textsf{crs}_{\textsf{P}}$ (needed by the prover) from $\textsf{crs}_{\textsf{V}}$ (needed by the verifier).
- Prover: $\textsf{P}$ is a PPT algorithm that, given $(\textbf{R}, \textsf{z}_{\textbf{R}}, \textsf{crs}_{\textsf{P}}, \vec {x}, \vec {w})$, where $(\vec {x}, \vec {w}) \in \textbf{R}$, outputs an argument $\pi$. Otherwise, it outputs $\bot$.
- Verifier: $\textsf{V}$ is a PPT algorithm that, given $(\textbf{R}, \textsf{z}_{\textbf{R}}, \textsf{crs}_{\textsf{V}}, \vec {x}, \pi )$, returns either 0 (reject) or 1 (accept).
- Simulator: $\textsf{Sim}$ is a PPT algorithm that, given $(\textbf{R}, \textsf{z}_{\textbf{R}}, \textsf{crs}, \vec {\textsf{ts}}, \ \vec {x})$, outputs a simulated argument $\pi$.

Besides *succinct* proofs, i.e. polynomial in $\lambda$, an SE zk-SNARK is required to satisfy *completeness*, *simulation extractability*, and *zero-knowledge*.

#### Definition 1

(*Perfect Completeness*) A non-interactive argument $\Psi$ is *perfectly complete for* $\mathscr {R}$, if for all $\lambda$, all $(\textbf{R}, \textsf{z}_{\textbf{R}}) \in \textbf{Im}(\mathscr {R}(1^\lambda ))$, and $(\vec {x}, \vec {w}) \in \textbf{R}$,

$$\begin{aligned} \Pr \left[ \begin{aligned}&\textsf{crs}\leftarrow \textsf{K}(\textbf{R}, \textsf{z}_{\textbf{R}}), \ \pi \leftarrow \textsf{P}(\textbf{R}, \textsf{z}_{\textbf{R}}, \textsf{crs}, \vec {x}, \vec {w}): \\ {}&\textsf{V}(\textbf{R}, \textsf{z}_{\textbf{R}}, \textsf{crs}, \vec {x}, \pi ) = 1 \end{aligned} \right] = 1. \end{aligned}$$

Intuitively, perfect completeness states that an honest prover $\textsf{P}$ always convinces an honest verifier $\textsf{V}$.

#### Definition 2

(*Computationally Knowledge-Soundness* [22]) A non-interactive argument $\Psi$ is computationally (adaptively) *knowledge-sound for *$\mathscr {R}$, if for every non-uniform PPT $\mathscr {A}$, there exists a non-uniform PPT extractor $\textsf{Ext}_\mathscr {A}$, s.t. for all $\lambda$, the following probability is $\textsf{negl}(\lambda )$,

$$\begin{aligned} \Pr \left[ \begin{aligned}&(\textbf{R}, \textsf{z}_{\textbf{R}}) \leftarrow \mathscr {R}(1^{\lambda }), (\textsf{crs}\,\Vert \,\vec {\textsf{ts}}) \leftarrow \textsf{K}(\textbf{R}, \textsf{z}_{\textbf{R}}), \\ {}&(\vec {x}, \pi ) \leftarrow \mathscr {A}(\textbf{R}, \textsf{z}_{\textbf{R}}, \textsf{crs}), \vec {w}\leftarrow \textsf{Ext}_\mathscr {A}(\textsf{trans}_\mathscr {A}):\\&(\vec {x}, \vec {w}) \not \in \textbf{R}\wedge \textsf{V}(\textbf{R}, \textsf{z}_{\textbf{R}}, \textsf{crs}, \vec {x}, \pi ) = 1 \end{aligned} \right] \hspace{5.0pt}. \end{aligned}$$

Here, $\textsf{trans}_\mathscr {A}$ is a list containing all of $\mathscr {A}$’s inputs and outputs. Intuitively, the definition states that if an adversary can convince the verifier, she *knows* the witness. A knowledge-sound $\Psi$ also is called an *argument of knowledge*.

#### Definition 3

(*Weak Simulation Extractability* [27]) A non-interactive argument $\Psi$ is *(non-black-box) weak simulation-extractable for* $\mathscr {R}$, if for any non-uniform PPT $\mathscr {A}$, there exists a non-uniform PPT extractor $\textsf{Ext}_\mathscr {A}$ s.t. for all $\lambda$, the following probability is $\textsf{negl}(\lambda )$,

$$\begin{aligned} \Pr \left[ \begin{aligned}&(\textbf{R}, \textsf{z}_{\textbf{R}}) \leftarrow \mathscr {R}(1^{\lambda }), (\textsf{crs}\,\Vert \,\vec {\textsf{ts}}) \leftarrow \textsf{K}(\textbf{R}, \textsf{z}_{\textbf{R}}), \\ {}&(\vec {x}, \pi ) \leftarrow \mathscr {A}^{\textsf{O}(\vec {\textsf{ts}},.)} (\textbf{R}, \textsf{z}_{\textbf{R}}, \textsf{crs}), \vec {w}\leftarrow \textsf{Ext}_\mathscr {A}(\textsf{trans}_\mathscr {A}): \\&\vec {x}\not \in Q \wedge (\vec {x}, \vec {w}) \not \in \textbf{R}\wedge \textsf{V}(\textbf{R}, \textsf{z}_{\textbf{R}}, \textsf{crs}, \vec {x}, \pi ) = 1 \end{aligned} \right] . \end{aligned}$$

Here, *Q* is the set of statements queried by adversary to the simulation oracle $\textsf{O}$, and $\textsf{trans}_\mathscr {A}$ is a list containing all of $\mathscr {A}$’s inputs and outputs. Note that this variant of simulation extractability allows proof randomization, while it ensures that a proof cannot be changed to prove a new statement.

#### Definition 4

(*Simulation Extractability* [23]) A non-interactive argument $\Psi$ is *(non-black-box strong) simulation-extractable for *$\mathscr {R}$, if for any non-uniform PPT $\mathscr {A}$, there exists a non-uniform PPT extractor $\textsf{Ext}_\mathscr {A}$ s.t. for all $\lambda$, the following probability is $\textsf{negl}(\lambda )$,

$$\begin{aligned} \Pr \left[ \begin{aligned}&(\textbf{R}, \textsf{z}_{\textbf{R}}) \leftarrow \mathscr {R}(1^{\lambda }), (\textsf{crs}\,\Vert \,\vec {\textsf{ts}}) \leftarrow \textsf{K}(\textbf{R}, \textsf{z}_{\textbf{R}}), \\ {}&(\vec {x}, \pi ) \leftarrow \mathscr {A}^{\textsf{O}(\vec {\textsf{ts}},.)} (\textbf{R}, \textsf{z}_{\textbf{R}}, \textsf{crs}), \vec {w}\leftarrow \textsf{Ext}_\mathscr {A}(\textsf{trans}_\mathscr {A}): \\&(\vec {x}, \pi ) \not \in Q \wedge (\vec {x}, \vec {w}) \not \in \textbf{R}\wedge \textsf{V}(\textbf{R}, \textsf{z}_{\textbf{R}}, \textsf{crs}, \vec {x}, \pi ) = 1 \end{aligned} \right] . \end{aligned}$$

Here, *Q* is the set of simulated statement-proof pairs generated by adversary’s queries to the simulation oracle $\textsf{O}$, and $\textsf{trans}_\mathscr {A}$ is a list containing all of $\mathscr {A}$’s inputs and outputs.

Note that both variants of *simulation extractability* implies *knowledge soundness* (given in Def. 2), as the earlier is a strong notion of the later which additionally the adversary is allowed to send query to the proof simulation oracle.

#### Definition 5

(*Zero-Knowledge (ZK)* [22]) A non-interactive argument $\Psi$ is *computationally ZK for* $\mathscr {R}$, if for all $\lambda$, all $(\textbf{R}, \textsf{z}_{\textbf{R}}) \in \textbf{Im}(\mathscr {R}(1^\lambda ))$, and for all non-uniform PPT $\mathscr {A}$, $\varepsilon _0 \approx _c \varepsilon _1$, where

$$\begin{aligned} \varepsilon _b = \Pr [(\textsf{crs}\,\Vert \,\vec {\textsf{ts}}) \leftarrow \textsf{K}(\textbf{R}, \textsf{z}_{\textbf{R}}): \mathscr {A}^{\textsf{O}_b (\cdot , \cdot )} (\textbf{R}, \textsf{z}_{\textbf{R}}, \textsf{crs}) = 1]. \end{aligned}$$

Here, the oracle $\textsf{O}_0 (\vec {x}, \vec {w})$ returns $\bot$ (reject) if $(\vec {x}, \vec {w}) \not \in \textbf{R}$, and otherwise it returns $\textsf{P}(\textbf{R}, \textsf{z}_{\textbf{R}}, \textsf{crs}_{\textsf{P}}, \vec {x}, \vec {w})$. Similarly, $\textsf{O}_1 (\vec {x}, \vec {w})$ returns $\bot$ (reject) if $(\vec {x}, \vec {w}) \not \in \textbf{R}$, otherwise it returns $\textsf{Sim}(\textbf{R}, \textsf{z}_{\textbf{R}}, \textsf{crs}, \vec {\textsf{ts}}, \vec {x})$. $\Psi$ is *perfect ZK for *$\mathscr {R}$ if one requires that $\varepsilon _0 = \varepsilon _1$.

Intuitively, a non-interactive argument is ZK if it does not leak extra information beyond the truth of the statement.

### Boneh-Boyen signatures

We briefly recall one of the constructions of Boneh-Boyen signatures [12], that is used implicitly in our constructions. Let $\mathbb {G}_1,\mathbb {G}_2,\mathbb {G}_T, e:\mathbb {G}_1\times \mathbb {G}_2\rightarrow \mathbb {G}_T$ be a bilinear group. Messages are elements of $\mathbb {Z}_p$, and signatures are elements of $\mathbb {G}_1$. The secret key is $\textsf{sk}\in \mathbb {Z}_p$, and the public key (verification key) is $[\textsf{sk}]_2\in \mathbb {G}_2$. To sign a message $m \in \mathbb {Z}_p$, the signer computes

$$\begin{aligned}_1=\left[ \frac{1}{\textsf{sk}+m}\right] _1. \end{aligned}$$

The verifier accepts the signature if the equation $e([\sigma ]_1,[\textsf{sk}]_2+[m]_2)=[1]_T$ holds.

Boneh-Boyen signatures are existentially unforgeable under the *q*-SDH assumption. We use them in our constructions as proofs of knowledge of the secret key in the AGM.

### Algebraic group model

The algebraic group model or AGM for short [19] assumes that adversaries are algebraic, i.e. they construct their output group elements as a linear combination of previously seen group elements. This model is a weakening of the GGM, [31, 33], since algebraic adversaries have direct access to group elements and can use their representation. In the asymmetric algebraic group model, it is assumed that, for every element $\pi$ in $\mathbb {G}_1,\mathbb {G}_2$ output by the adversary, it also outputs a set of coefficients in the field that express $\pi$ as a linear combination of previously received group elements in the same source group. For elements in $\mathbb {G}_T$, the adversary also outputs the coefficients that express every element output by the adversary as a linear combination of elements in $\mathbb {G}_T$ that the adversary has received or can compute as the pairing of elements in $\mathbb {G}_1$ and $\mathbb {G}_2$ it has received.

Several works (e.g. [17]) have proven security in the AGM with random oracles. In this case, the adversary has oracle access to a certain function $H: \{0,1\}^* \rightarrow R$, and the assumption is that for every element $\pi$ output by the adversary in $\mathbb {G}_1,\mathbb {G}_2$, also outputs a set of coefficients in the field that express $\pi$ as a linear combination of all previously received group elements, including those obtained as a response to a hash query if the range *R* is a group.

Note that when the range of the hash function *R* is a group, the oracle allows the adversary to sample obliviously from it, i.e. without knowing the discrete logarithm. In our case, the range of the RO is a field (of size of the order of the elliptic curve) and therefore in our model, the adversary cannot obliviously sample in the group. As discussed by Lipmaa [29], we could consider strengthening our model and give the adversary access to another oracle $H_2$ mapping to group elements to give this additional power to the adversary. This model is more realistic since in practice there usually exist hash to group algorithms that allow to sample in the curve without knowing the discrete logarithm.

Although the strengthened model is very meaningful and is a more realistic idealization of elliptic curves, we have not considered since it complicates the proof significantly although these additional uniformly and randomly chosen elements that are chosen independently of the input of the adversary, intuitively, cannot help the adversary except with negligible probability.^2^

Following the work of Fuchsbauer et al. [19], we will prove that the security of our scheme reduces to the $(q_1,q_2)$-DLOG Assumption, for a certain $(q_1,q_2)$ that depends on the size of supported instances. We note that to improve efficiency, as [29] we rely on the asymmetric AGM, as opposed to the proof of $\textsf{Groth16}$ in [19, 22].

#### Definition 6

The $(q_1,q_2)$-*DLOG* Assumption holds relative to $\textsf{BGgen}(1^{\lambda })$ if for all PPT adversaries $\mathscr {A}$, the following probability is $\textsf{negl}(\lambda )$,

$$\begin{aligned} \Pr \left[ \begin{aligned}&\textsf{gk}\leftarrow \textsf{BGgen}(1^{\lambda }), \ z\leftarrow \mathbb {Z}_p: \\ {}&z \leftarrow \mathscr {A}\left( \textsf{gk},\left\{ [z^i]_1\right\} _{i=0}^{q_1},\left\{ [z^i]_2\right\} _{i=0}^{q_2}\right) \end{aligned} \right] . \end{aligned}$$

## SE variant of $\textsf{Groth16}$ in the ROM

To achieve (strong) simulation extractability, the prover of Bowe and Gabizon’s construction [14] replaces all the computations which depend on $\delta$ given in the $\textsf{crs}$ by some $\delta '$ of its choice, that it must give as part of the proof, together with a proof of knowledge of the DLOG of $\delta '$ w.r.t to $\delta$, which given some element $[Y]_1=H([A]_1 \parallel [B]_2 \parallel [C]_1 \parallel [\delta ']_2)$, consists of $[\pi ]_1$ such that $e([Y]_1,[\delta ']_2) = e([\pi ]_1,[\delta ]_2)$. In their analysis, *H* is an RO and their proof requires 2 pairings for verification.

**Fig. 1:**
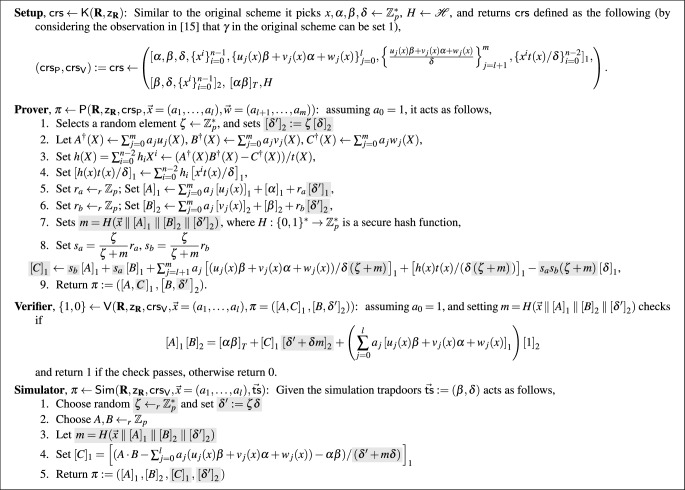
A simulation-extractable variation of $\textsf{Groth16}$ for $\textbf{R}$. $\mathscr {H}$ is a family of collision resistant hash functions that map to $\mathbb {Z}_p^*$

In Fig. 1, we describe a SE variant of $\textsf{Groth16}$ that uses a new technique to shorten the proof and verifies it with a single verification equation which requires 3 pairings, just as $\textsf{Groth16}$. The security analysis is done in the AGM assuming the underlying hash function is a random oracle. The SE proof is built using a sequence of games. As part of the reduction we need to rewrite in the AGM part of the same proof as Bowe and Gabizon’s construction [14], that is also in the random oracle but in the generic group model.

A part from the efficiency gain, from a security point of view one additional advantage of our construction is that the RO maps to elements in $\mathbb {Z}_p$ and it does not need the property that *H* can sample elements of $\mathbb {G}$ obliviously (i.e. soundness does not use that the DLOG of image elements is hard).

The idea of Bowe and Gabizon of using a POK of the DLOG of $\delta '$ was also used in our preliminary results presented in [7], included in Appendix A. The construction we present below improves on both previous works by choosing $\delta '$ as before but then replacing it by $\delta '+\delta m$ to create and verify the proof at once, where, $m:= H(\vec {x}\parallel \left[ {A}\right] _{1} \parallel \left[ {B}\right] _{2} \parallel \left[ \delta '\right] _{2})$. The intuition is that the adversary needs to know the division in the exponent of *C* by $\delta '+\delta m$. However, this is a degree one polynomial in $\delta$, and this is hard to do unless $\delta '=\zeta \delta$. The verification of this variant requires one additional exponentiation in $\mathbb {G}_2$. In the description of the new construction, we highlight the changes to $\textsf{Groth16}$ with  background. We emphasize that the original scheme corresponds to $m=0$ and $\zeta =1$.

### Theorem 1

(Completeness, ZK, strong SE) The variant of $\textsf{Groth16}$ described in Fig. 1, is a non-interactive zero-knowledge argument that guarantees 1) perfect completeness, 2) perfect zero-knowledge and 3) strong simulation-extractability in the asymmetric Generic Group Model and the RO Model.

### Proof

To see why perfect completeness holds, the easiest is to rewrite this scheme in such a way so that the terms *A*, *B*, *C* correspond exactly to $\textsf{Groth16}$, except that the original term $\delta$ is replaced by $\delta '+\delta m$. The prover creates *A*, *B* with the randomizer $r_a\delta ',r_b \delta '$, $r_a,r_b \leftarrow \mathbb {Z}_p$. Then, it receives *m* and reinterprets *A*, *B* as being created for the randomized $\delta '+\delta m$ and some random values $s_a,s_b$. This means the prover finds the value $s_a$ such that $r_a \delta ' = s_a (\delta '+\delta m)$. Solving the equation, we get $s_a = \dfrac{\zeta }{\zeta + m} r_a$ (similarly, $s_b = \dfrac{\zeta }{\zeta + m} r_b$). Then it computes *C* as in the original $\textsf{Groth16}$ paper but for $s_a,s_b$ and $\delta '+\delta m$, instead of $\delta$. Rewriting, we obtain:

$$\begin{aligned}{} & {} \left[ {A}\right] _{1} \leftarrow \sum _{j = 0}^m a_j \left[ u_j (x)\right] _{1} + \left[ \alpha \right] _{1} + s_a \left[ \delta '+ \delta m\right] _{1},\\{} & {} \left[ {B}\right] _{2} \leftarrow \sum _{j = 0}^m a_j \left[ v_j (x)\right] _{2} + \left[ \beta \right] _{2} + s_b \left[ \delta '+\delta m\right] _{2},\\{} & {} \left[ {C}\right] _{1} \leftarrow s_b \left[ {A}\right] _{1} +s_a \left[ {B}\right] _{1} \\{} & {} \quad + \sum _{j = l + 1}^m a_j \left[ (u_j (x) \beta + v_j (x) \alpha + w_j (x)) /(\delta '+\delta m)\right] _{1} \\{} & {} \quad + \left[ h(x) t(x) / (\delta '+\delta m)\right] _{1} - s_a s_b \left[ \delta '+ \delta m \right] _{1}. \end{aligned}$$

Completeness easily follows from these formulae (in fact, it is identical to the completeness of $\textsf{Groth16}$ replacing $\delta$ by $\delta '+\delta m$). Similarly, perfect zero-knowledge can be argued in a standard way.

Simulation extractability is proven by reduction in the AGM to the knowledge soundness of $\textsf{Groth16}$.

Since the adversary is algebraic, for each output elements it is possible to extract a list of coefficients that express it as a linear combination of previously seen elements. The view of an adversary $\mathscr {A}$ that has made a sequence of queries $\vec {x}_1,\dots ,\vec {x}_v$ to $\textsf{Sim}(\vec {\textsf{ts}},\cdot )$, and received answers $\{\pi _j=([{A}_j, {C}_j]_1, [{B}_j, \delta _j]_2)\}_{j=1}^v$ is the set $Q'$, the union of elements in the $\textsf{crs}$ together with those from the replies of $\textsf{Sim}(\vec {\textsf{ts}},\cdot )$; namely,

$$\begin{aligned} Q' :=&\left( \begin{aligned}&\textstyle \ \left[ \alpha , \beta , \delta , \{x^i\}_{i = 0}^{n-1},\right. \{u_j (x) \beta + v_j (x) \alpha + w_j (x)\}_{j = 0}^{l}, \\ {}&\left\{ \frac{u_j (x) \beta + v_j (x) \alpha + w_j (x)}{\delta }\right\} _{j = l + 1}^m , \textstyle \left. \{x^i t(x) / \delta \}_{i = 0}^{n - 2} \right] _1,\\ {}&[ \beta , \delta , \{x^i\}_{i = 0}^{n-1}]_2 \end{aligned} \right) \\ {}&\hspace{5mm} \cup \left( \begin{aligned}&\textstyle \left\{ \left[ A_j, C_j :=\frac{A_j B_j - \textsf {ic}_j - \alpha \beta }{\delta _j+m_j\delta },\right] _1 , \textstyle [B_j , \delta _j]_2, m_j\right\} _{j = 1}^{v} \end{aligned} \right) \end{aligned}$$

where $\textsf {ic}_j=\sum _{i = 0}^{l} a_i^j (u_i (x) \beta + v_i (x) \alpha + w_i (x))$, $\vec x_j=(a_1^j,\dots ,a_l^j)$, and $m_j\in \mathbb {Z}_p$ the message that simulator receives from the RO for each $A_j,B_j,\delta _j$. Let $Q_1'$ be the elements of $Q'$ in group $\mathbb {G}_1$ and $Q_2'$ the elements in group $\mathbb {G}_2$.

Now, assume that the adversary $\mathscr {A}$ has produced elements $\pi =([{A}, {C}]_1, [{B}, \delta ']_2)$ that pass the verification equation. This implies that $C=({A}{B}-\alpha \beta - \sum _{j=0}^{l} a_j (u_j (x) \beta + v_j (x) \alpha + w_j (x)))/(\delta '+m\delta )$, where $m = H(\vec {x}\parallel [A]_1 \parallel [B]_2 \parallel [\delta ']_2)$. The coefficients extracted for output element $[Y]_i$ for $i \in \{1,2\}$ corresponding to element $q \in Q_i'$ will be denoted by $k_{Y,q}$, so that for each element *Y* we have that $Y=\sum _{q \in Q_i'} k_{Y,q} q$.

The reduction proceeds in a series of games, $G0,\ldots ,G4$.

G0: This is the original simulation extractability soundness game. The adversary wins if the proof $\pi =(\left[ {A}, {C}\right] _{1}, \left[ {B}, \delta '\right] _{2})$ for some statement $(a_1,\ldots ,a_l)$ is accepted and it is not the result of some previous query for the same statement.
G1: This game is the same as the previous one except that it aborts if $\pi$ is accepted but $k_{\delta ',\delta }=-m$.
G2: This game is the same as the previous one except that it aborts if $\pi$ is accepted but for some $j=1,\ldots ,v$, $\delta '=k_{\delta ',\delta _j} \delta _j+k_{\delta ',\delta } \delta$ and $m=m_j k_{\delta ',\delta _j}-k_{\delta ',\delta }$.
G3: This game is the same as the previous one except that it aborts if if $\pi$ is accepted but $\delta ' \ne k_{\delta ',\delta } \delta$.
G4: This game is the same as the previous one, except that an abort occurs if $\pi$ is accepted but to compute $\pi$ the adversary uses any of the answers of the simulation oracle.

From G3 on, it is clear that the reduction can extract $\zeta =DLOG_{\delta }\ \delta '$ from the adversary, from which it can transform the adversary’s output to a proof for $\textsf{Groth16}$ as $[A]_1, [B]_2, [C(\zeta +m)]_1$. Additionally, since in G4 the adversary does not use any of the answers to the simulation oracle, soundness in that game is implied by the knowledge soundness of $\textsf{Groth16}$.

We now proceed to bound the difference in the advantage in these games of any algebraic adversary $\mathscr {A}$. Clearly, $|\Pr [\text {G0}(\mathscr {A})=1]-Pr[\text {G1}(\mathscr {A})=1]|=|\Pr [\text {G1}(\mathscr {A})=1]-Pr[\text {G2}(\mathscr {A})=1]|=1/p$ since the output of the random oracle is a uniform value chosen independently of the constants extracted, and the adversary can only be lucky in guessing this value with probability 1/*p*.

Next we prove the following lemma:

### Lemma 1

For all PPT algebraic adversaries $\mathscr {A}$ there exists an adversary $\mathscr {B}$ against the $(v+2,1)$-DLOG Assumption such that

$$\begin{aligned} Pr[\text {G2}(\mathscr {A})=1] \le Pr[\text {G3}(\mathscr {A})=1]+ \textsf{Adv}_{\mathscr {B}}(\lambda )+ \textsf{negl}(\lambda )\end{aligned}$$

### Proof

Both games are identical except if adversary $\mathscr {A}$ outputs $\delta '\ne k_{\delta ',\delta } \delta$. We show that in this case there exists another adversary $\mathscr {B}$ that breaks the $(v+2,1)$-DLOG Assumption.

Given some group key $\textsf{gk}'=(p,\mathbb {G}_1,\mathbb {G}_2,\mathbb {G}_T,e,\mathscr {P}'_1, \mathscr {P}_2) \leftarrow \textsf{BGgen}(1^{\lambda })$, adversary $\mathscr {B}$ receives $\left\{ z^i \mathscr {P}_1'\right\} _{i=0}^{v+2},\left\{ z^i\mathscr {P}_2\right\} _{i=0}^{1}$. It then chooses $m_1,\ldots ,m_v$ random values in $\mathbb {Z}_p$. It will store these values and give them as a reply to the hash queries related to the simulation queries of $\mathscr {A}$. Next, for $j=1,\ldots ,v$, it defines

$$\begin{aligned} \delta _j= d_j z+f_j, \ \ f_j,d_j \leftarrow \mathbb {Z}_p \ \ \text {and} \ \ \delta =dz+f,\ \ f,d \leftarrow \mathbb {Z}_p. \end{aligned}$$

It programs the public parameters to compute $\delta$ and $\delta _j+m_j \delta$ roots for any *j*, that is, it defines the new group key included in the public parameters to be $\textsf{gk}=(p,\mathbb {G}_1,\mathbb {G}_2,\mathbb {G}_T,e,\mathscr {P}_1= \delta \prod _{j=1}^v (\delta _j+m_j\delta )\mathscr {P}'_1,\mathscr {P}_2)$. This can be computed from the input of $\mathscr {B}$ since

$$\begin{aligned} \delta \prod _{j=1}^v (\delta _j+m_j\delta ){} & {} =(dz+f) \prod _{j=1}^v\\{} & {} \qquad \times ((d_j+m_jd)z+(f_j+m_j f)) \end{aligned}$$

is a polynomial of degree $(v+1)$ in the indeterminate *z*.

Then, adversary $\mathscr {B}$ samples $x,\alpha ,\beta \leftarrow \mathbb {Z}_p$ and computes the common reference string honestly based on the new group key $\textsf{gk}$ and sends all this information to $\mathscr {A}$. Note that this requires to compute some expressions involving $x,\alpha ,\beta$ divided by $\delta$ but $\mathscr {B}$ can do that by computing $\delta ^{-1}\mathscr {P}_1$, which is $\prod _{j=1}^v (\delta _j+m_j\delta )\mathscr {P}_1'=\prod _{j=1}^v (d_j z+f_j+m_j) \mathscr {P}_1$. The terms in $\mathbb {G}_1$ have maximal degree $v+2$ so they can be computed by $\mathscr {B}$. Whenever $\mathscr {B}$ receives a simulation query $\vec x_j$, it sets $[{A}_j]_{1}=[\alpha ]_{1}+r_{a_j}[\delta _j+m_j\delta ]_{1}$ and $[B_j]_{2}=[\beta ]_{2}+r_{b_j}[\delta _j+m_j \delta ]_{2}$, declares $H(\vec {x}\parallel [A]_1 \parallel [B]_2 \parallel [\delta _j])$ and computes

$$\begin{aligned}{}[C_j]_1=\left[ \frac{A_j B_j-\textsf{ic}_j-\alpha \beta }{\delta _j+m_j\delta }\right] _1. \end{aligned}$$

For this, it will use the fact that it can compute $(\delta _j+m_j \delta )^{-1}\mathscr {P}_1$ as $(dz+f) \prod _{i=1,i \ne j}^v (d_i z+f_i+m_i)\mathscr {P}_1'$.

If adversary $\mathscr {A}$ breaks simulation extractability for some $\vec x=(a_1,\ldots ,a_j)$, it has produced elements $(A, B, C, \delta ')$ that pass the verification equation so:

1 $$\begin{aligned} C=\frac{A B-\textsf{ic}-\alpha \beta }{\delta '+m\delta }.\end{aligned}$$

We now study the denominator and numerator of this expression.

For a second consider $\vec \Delta =(\delta , \delta _1,\ldots , \delta _v)$ as formal variables and define the polynomial

$$\begin{aligned}{} & {} P_{\delta '}(\vec \Delta )= k_{\delta ',1} +k_{\delta ',\beta }\beta + k_{\delta ',\delta }\delta + \sum _{i=0}^{n-1} k_{\delta ',x^i} x^{i} \\{} & {} \quad \quad \quad + \sum _{j=1}^v ( k_{\delta ',B_j} B_j+k_{\delta ',\delta _j} \delta _j). \end{aligned}$$

The polynomial $P_{B}(\vec \Delta )$ is defined analogously for the coefficients $k_{B,q}$, with $q \in Q'_2$. On the other hand, we also define $R_{A}(\vec \Delta )$, $R_{C}(\vec \Delta )$ in a similar way, except that the result is not a polynomial but a sum of some rational functions since the view of $\mathscr {A}$ in $\mathbb {G}_1$ includes terms that have $\delta ,\delta _j+m_j \delta$ in the denominator.

If adversary $\mathscr {A}$ successfully distinguishes between the two games, $k_{\delta ',\delta } \ne -m$, so $P_{\delta '}(\vec \Delta )+ m \delta$ is a polynomial of degree one in $\delta$. Further, there is no *j* such that $P_{\delta '}(\vec \Delta )+m\delta = \chi (\delta _j+m_j\delta )$ for some $\chi \in \mathbb {Z}_p$, since this would imply $\delta '=k_{\delta ',\delta _j} \delta _j+k_{\delta ',\delta } \delta$ and $m=m_j k_{\delta ',\delta _j}-k_{\delta ',\delta }$, which is also an abort condition. If $\mathscr {A}$ is successful in distinguishing between the two games, $P_{\delta '}(\vec \Delta )\ne k_{\delta ',\delta } \delta$, and we are left with two possibilities:

- $$\begin{aligned} R_C(\vec \Delta ) = \dfrac{R_{A}(\vec \Delta )P_{B}(\vec \Delta )- \textsf{ic}-\alpha \beta }{P_{\delta '}(\vec \Delta )+m \delta }. \end{aligned}$$
  But this equation cannot hold, since as we argued, $P_{\delta '}(\vec \Delta )+m \delta$ is not a polynomial that is a multiple of $\delta$, or $\delta _j+m_j \delta$, the only terms that appear as denominators in any term in $R_C(\vec \Delta )$.
- otherwise,
  $$\begin{aligned} R_C(\vec \Delta )(P_{\delta '}(\vec \Delta )+m \delta ) - R_{A}(\vec \Delta ) P_{B}(\vec \Delta )+ \textsf{ic}+\alpha \beta \ne 0. \end{aligned}$$
  Define
  $$\begin{aligned}{} & {} T(\vec \Delta )=\delta \prod _{j=1}^v (\delta _j+m_j \delta )\Big (R_C(\vec \Delta )(P_{\delta '}(\vec \Delta )+m \delta )\\ {}{} & {} \quad - R_{A}(\vec \Delta ) P_{B}(\vec \Delta )+ \textsf{ic}+\alpha \beta \Big ). \end{aligned}$$
  Note that this is a polynomial in $\vec \Delta$, since $\delta \prod _{j=1}^v (\delta _j+m_j \delta )$ cancels out any of the denominators that appear in the terms in $R_A(\vec \delta )$. Replacing $\delta =dZ+f$ and $\delta _j= d_j Z+ f_j$ in *T* we get a polynomial that depends on a single variable $T'(Z)$. Since $C=\frac{A B-\textsf{ic}-\alpha \beta }{\delta '+m\delta }$, $T'(z)=0$. On the other hand, $T'(Z)\ne 0$ except with probability 1/*p*. This is justified as follows: if $T'(Z)$ was 0 all its coefficients must be 0. In particular, take the leading terms in *Z* of $T'(Z)$: this is an expression involving only $d,d_j$, which are information theoretically hidden from $\mathscr {A}$. If we think of this polynomial as a multivariate one of total degree $v+3$ in variables $d,d_j$, the probability that $\mathscr {A}$ chooses the coefficients $k_{A,q}, k_{B,q},k_{\delta ',q},k_{C,q}$ such that when evaluated in $d,d_j$ this polynomial is 0 can be bounded by $(v+3)/p$. Therefore, $\mathscr {B}$ can solve the DLOG challenge by factoring $T'$ and trying all the possible roots.

### Lemma 2

For all PPT algebraic adversaries $\mathscr {A}$ there exists an adversary $\mathscr {B}$ against the $(v+2,1)$-DLOG Assumption such that

$$\begin{aligned} Pr[\text {G3}(\mathscr {A})=1] \le Pr[\text {G4}(\mathscr {A})=1]+ \textsf{Adv}_{\mathscr {B}}(\lambda )+ \textsf{negl}(\lambda )\end{aligned}$$

### Proof

Both games are identical except if adversary $\mathscr {A}$ outputs a accepting proof that is built using the output of some simulation query. We show that in this case there exists another adversary $\mathscr {B}$ that breaks the $(v+2,1)$-DLOG Assumption.

Given some group key $\textsf{gk}'=(p,\mathbb {G}_1,\mathbb {G}_2,\mathbb {G}_T,e,\mathscr {P}'_1, \mathscr {P}_2) \leftarrow \textsf{BGgen}(1^{\lambda })$, adversary $\mathscr {B}$ receives $\left\{ z^i \mathscr {P}_1'\right\} _{i=0}^{v+1},\left\{ z^i\mathscr {P}_2\right\} _{i=0}^{1}$. It then chooses $m_1,\ldots ,m_v$ random values in $\mathbb {Z}_p$. It will store these values and give them as a reply to the hash queries related to the simulation queries of $\mathscr {A}$. Next, for $j=1,\ldots ,v$, it defines

$$\begin{aligned} \alpha = d_{\alpha } z+f_{\alpha }, \ f_{\alpha },d_{\alpha }\leftarrow \mathbb {Z}_p \qquad \beta = d_{\beta } z+f_{\beta }, \ f_{\beta },d_{\beta } \leftarrow \mathbb {Z}_p, \end{aligned}$$

and, as in the previous lemma:

$$\begin{aligned} \delta _j= d_j z+f_j, \ \ f_j,d_j \leftarrow \mathbb {Z}_p \ \ \text {and} \ \ \delta =dz+f,\ \ f,d \leftarrow \mathbb {Z}_p. \end{aligned}$$

It programs the public parameters to compute $\delta$ and $\delta _j+m_j \delta$ roots for any *j*, that is, it defines the new group key included in the public parameters to be $\textsf{gk}=(p,\mathbb {G}_1,\mathbb {G}_2,\mathbb {G}_T,e,\mathscr {P}_1= \delta \prod _{j=1}^v (\delta _j+m_j\delta )\mathscr {P}'_1,\mathscr {P}_2)$. This can be computed from the input of $\mathscr {B}$ since

$$\begin{aligned} \delta \prod _{j=1}^v (\delta _j+m_j\delta )=(dz+f) \prod _{j=1}^v ((d_j+m_jd)z+(f_j+m_j f)) \end{aligned}$$

is a polynomial of degree $(v+1)$ in the indeterminate *z*.

Then, adversary $\mathscr {B}$ samples $x \leftarrow \mathbb {Z}_p$ and computes the common reference string honestly based on the new group key $\textsf{gk}$ and sends all this information to $\mathscr {A}$. This can be computed from $\mathscr {B}$’s input since these requires to compute polynomials of degree at most 2 in *z* in each source group.

Whenever $\mathscr {B}$ receives a simulation query $\vec x_j$, it samples $\zeta _j, f_{A,j},d_{A,j},f_{B,j},d_{B,j} \leftarrow \mathbb {Z}_p$, and sets

$$\begin{aligned} A_j=d_{A,j}z+f_{A,j} \qquad B_j=d_{B,j}z+f_{B,j} \qquad \delta _j=\zeta _j \delta , \end{aligned}$$

declares $m_j=H(\vec {x}_j \parallel [A_j]_1\parallel [B_j]_2\parallel [\delta _j])$ and computes

$$\begin{aligned}{}[C_j]_1=\left[ \frac{A_j B_j-\textsf{ic}_j-\alpha \beta }{\delta _j+m_j\delta }\right] _1. \end{aligned}$$

For this, it uses the fact that it can compute $\delta _j+m_j\delta _j$ roots in $\mathbb {G}_1$. If adversary $\mathscr {A}$ distinguishes between both games, it outputs some $\vec x=(a_1,\ldots ,a_j)$, and $(A, B, C, \delta ')$ that pass the verification equation and, further, it is possible to extract some $\zeta$ such that $\delta '+m\delta =(\zeta +m) \delta$, therefore it holds that:

2 $$\begin{aligned} C\delta (\zeta +m)- A B+\textsf{ic}+\alpha \beta =0.\end{aligned}$$

For a second, consider

$$\begin{aligned} \vec Y:=(\alpha ,\beta ,\delta ,\delta _1,\ldots ,\delta _v, A_1,\ldots ,A_v,B_1,\ldots ,B_v) \end{aligned}$$

as formal variables. Define the polynomial

$$\begin{aligned} P_{B}(\vec Y)= & {} k_{B,1} +k_{B,\beta }\beta + k_{B,\delta }\delta + \sum _{i=0}^{n-1} k_{B,x^i} x^{i} \\{} & {} \quad + \sum _{j=1}^v ( k_{B,B_j} B_j+k_{B,\delta _j}. \delta _j). \end{aligned}$$

Define $R_{A}(\vec Y)$, $R_{C}(\vec Y)$ in a similar way, with the coefficients $k_{A,q},k_{C,q}$, $q \in Q'_1$ extracted from the adversary, except that the result is not a polynomial but a sum of some rational functions since the view of $\mathscr {A}$ in $\mathbb {G}_1$ includes terms that have $\delta$ or $\delta _j+m_j \delta$ in the denominator. Note that $P_{A}(\vec Y):=\delta \prod _{j=1}^v (\delta _j+m_j \delta )R_{A}(\vec Y)$, $P_{C}(\vec Y):= \delta \prod _{j=1}^v (\delta _j+m_j \delta )R_{A}(\vec Y)$ are polynomials in $\vec Y$ of degree $v+2$ since all possible denominators are cancelled out. Multiplying on both sides of equation (2) by $\delta \prod _{j=1}^v (\delta _j+m_j \delta )$, and replacing each group element by the corresponding polynomial, we get the following polynomial:

3 $$\begin{aligned} T(\vec Y)= & {} P_C(\vec Y)(\zeta +m)\delta - P_A(\vec Y) P_B(\vec Y)\nonumber \\{} & {} \quad +\delta \prod _{j=1}^v (\delta _j+m_j \delta ) \textsf{ic}(\vec Y) +\delta \prod _{j=1}^v (\delta _j+m_j \delta ) \alpha \beta .\nonumber \\ \end{aligned}$$

If adversary $\mathscr {A}$ distinguishes between the two games, there is at least one coefficient of $P_C(\vec Y)$ or $P_A(\vec Y)$ accompanying $A_j$ or $C_j$ which is not zero, or at least one coefficient of $P_B(\vec Y)$ accompanying $\delta _j$ or $B_j$ which is not zero. We show that this implies in all cases that $T(\vec Y) \ne 0$.

We start by arguing that $k_{A, \alpha }=1$ and $k_{B,\beta }=1$, since otherwise the term $\alpha \beta$ in equation (3) cannot be cancelled out. In other words, $R_A(\vec Y)=\alpha +\ldots$ and $P_B(\vec Y)=\beta + \ldots$, so $P_A(\vec Y)=\delta \prod _{j=1}^v (\delta _j+m_j \delta )\alpha +\ldots$. We next argue all cases of interest separately:

- If the coefficient $k_{B,\delta _j} \ne 0$ for some *j*, then in $P_A(\vec Y)P_B(\vec Y)$ the coefficient of $\alpha \delta \prod _{j=1}^v (\delta _j+m_j \delta )\delta _j$ is $k_{B,\delta _j}$ but it is 0 for the rest of the terms ($P_{C}$ can have no $\delta _j$ terms because the group is asymmetric, $\textsf{ic}(\vec Y)$ does not have $\delta _j$ terms by definition and the last term has no monomials without $\beta$). Therefore, the coefficient of this polynomial is not zero and $T'(\vec Y) \ne 0$.
- Similarly, if the coefficient $k_{B,B_j} \ne 0$ for some *j*, then in $P_A(\vec Y)P_B(\vec Y)$ the coefficient of $\alpha \delta \prod _{j=1}^v (\delta _j+m_j \delta )B_j$ is $k_{B,B_j}$, while in the other terms it is 0, in which case $T'(\vec Y) \ne 0$.
- If the coefficient $k_{A,A_j} \ne 0$ for some *j*, then in $P_A(\vec Y)P_B(\vec Y)$ the coefficient of monomial $A_j \beta$ is $k_{A,A_j}$, while in the other terms it is 0 (because in $P_{C}$ there can be no $\beta$ term and $\textsf{ic}(\vec Y)$ does not have $A_j$ terms by definition). Therefore, $T'(\vec Y) \ne 0$.
- If the coefficient $k_{A,C_j} \ne 0$ for some *j*, the analysis is the same as in (b). Therefore, $T'(\vec Y) \ne 0$.
- If the coefficient $k_{C,A_j} \ne 0$, the only term with $A_j$ would be $P_{C}(\vec Y)(\zeta _j+m)\delta$ since we ruled out case (c). Therefore, $T'(\vec Y) \ne 0$.
- If the coefficient $k_{C,C_j} \ne 0$, the only term with $C_j$ would be $P_{C}(\vec Y)(\zeta _j+m)\delta$ since we ruled out case (d). Therefore, $T'(\vec Y) \ne 0$.

Finally, we show that if $T(\vec Y) \ne 0$, there exists an adversary against the $(v+2,1)$-DLOG Assumption. Indeed, suppose that $T(\vec Y) \ne 0$. Define the univariate polynomial $T'(Z)$ as the result of substituting each variable in $\vec Y$ by an expression in the same indeterminate *Z*, as $\alpha = d_{\alpha } Z+f_{\alpha }, \beta = d_{\beta } Z+f_{\beta }, \delta _j= d_j Z+f_j, \delta =dZ+f$. If $T'(Z) \ne 0$ is not zero and we know from expression (2) that $T'(z)=0$, adversary $\mathscr {B}$ can find *z* by factoring $T'$, solving the DLOG challenge. On the other hand, to argue that $T'(Z) \ne 0$ except with probability $(v+3)/p$, we resort to the same argument as in the last step of Lemma 1.

This concludes the reduction to the knowledge soundness of $\textsf{Groth16}$, that was reduced in the symmetric AGM to the $(2n-1)$-DLOG Assumption.

## Empirical analysis

**Table 2:** A comparison of practical efficiency of our proposed variants of $\textsf{Groth16}$ along with the relevant SE zk-SNARKs for arithmetic circuit satisfiability

EC	zk-SNARK	SE	Model	PCPT (ns)	Proof Size (B)	Verifying 1 Proof	Ver. $10^2$ Proofs	Ver. $10^3$ Proofs
BLS12-381	Groth [6, 19, 22]	WSE	AGM	$\approx$ 5026	127.5	$\approx$ 1.90 ms	$\approx$ 0.190 s	$\approx$ 1.90 s
	Groth-Maller [23]	SSE	GGM	$\approx$ 11042	127.5	$\approx$ 3.32 ms	$\approx$ 0.332 s	$\approx$ 3.32 s
	Bowe-Gabizon [14]	SSE	GGM, RO	$\approx$ 5052	223.1	$\approx$ 3.52 ms	$\approx$ 0.352 s	$\approx$ 3.52 s
	BPR [7], Appendix A	SSE	GGM, CRH	$\approx$ 5042	223.1	$\approx$ 4.85 ms	$\approx$ 0.360 s	$\approx$ 3.50 s
	Sect. 3	SSE	AGM, RO	$\approx$ 5041	191.2	$\approx$ 2.39 ms	$\approx$ 0.194 s	$\approx$ 1.91 s
MNT4-298	Groth [6, 19, 22]	WSE	AGM	$\approx$ 4830	149.0	$\approx$ 2.67 ms	$\approx$ 0.267 s	$\approx$ 2.67 s
	Groth-Maller [23]	SSE	GGM	$\approx$ 10025	149.0	$\approx$ 3.80 ms	$\approx$ 0.380 s	$\approx$ 3.80 s
	Bowe-Gabizon [14]	SSE	GGM, RO	$\approx$ 4879	260.7	$\approx$ 4.32 ms	$\approx$ 0.432 s	$\approx$ 4.32 s
	BPR [7], Appendix A	SSE	GGM, CRH	$\approx$ 4881	260.7	$\approx$ 4.45 ms	$\approx$ 0.311 s	$\approx$ 3.05 s
	Sect. 3	SSE	AGM, RO	$\approx$ 4875	223.5	$\approx$ 3.33 ms	$\approx$ 0.271 s	$\approx$ 2.68 s
MNT6-298	Groth [6, 19, 22]	WSE	AGM	$\approx$ 5794	186.2	$\approx$ 4.94 ms	$\approx$ 0.494 s	$\approx$ 4.94 s
	Groth-Maller [23]	SSE	GGM	$\approx$ 11427	186.2	$\approx$ 7.07 ms	$\approx$ 0.707 s	$\approx$ 7.07 s
	Bowe-Gabizon [14]	SSE	GGM, RO	$\approx$ 5831	335.2	$\approx$ 8.07 ms	$\approx$ 0.807 s	$\approx$ 8.07 s
	BPR [7], Appendix A	SSE	GGM, CRH	$\approx$ 5824	335.2	$\approx$ 8.34 ms	$\approx$ 0.582 s	$\approx$ 5.72 s
	Sect. 3	SSE	AGM, RO	$\approx$ 5810	298.0	$\approx$ 6.11 ms	$\approx$ 0.501 s	$\approx$ 4.97 s
MNT4-753	Groth [6, 19, 22]	WSE	AGM	$\approx$ 30247	376.5	$\approx$ 29.1 ms	$\approx$ 2.91 s	$\approx$ 29.1 s
	Groth-Maller [23]	SSE	GGM	$\approx$ 83120	376.5	$\approx$ 41.6 ms	$\approx$ 4.16 s	$\approx$ 41.6 s
	Bowe-Gabizon [14]	SSE	GGM, RO	$\approx$ 30863	658.8	$\approx$ 47.3 ms	$\approx$ 4.73 s	$\approx$ 47.3 s
	BPR [7], Appendix A	SSE	GGM, CRH	$\approx$ 30887	658.8	$\approx$ 45.5 ms	$\approx$ 3.41 s	$\approx$ 33.8 s
	Sect. 3	SSE	AGM, RO	$\approx$ 30760	564.7	$\approx$ 33.9 ms	$\approx$ 2.94 s	$\approx$ 29.2 s
MNT6-753	Groth [6, 19, 22]	WSE	AGM	$\approx$ 33298	470.6	$\approx$ 53.6 ms	$\approx$ 5.36 s	$\approx$ 53.6 s
	Groth-Maller [23]	SSE	GGM	$\approx$ 83121	470.6	$\approx$ 76.9 ms	$\approx$ 7.69 s	$\approx$ 76.9 s
	Bowe-Gabizon [14]	SSE	GGM, RO	$\approx$ 33358	847.1	$\approx$ 88.5 ms	$\approx$ 8.85 s	$\approx$ 88.5 s
	BPR [7], Appendix A	SSE	GGM, CRH	$\approx$ 33359	847.1	$\approx$ 85.4 ms	$\approx$ 6.33 s	$\approx$ 63.1 s
	Sect. 3	SSE	AGM, RO	$\approx$ 33345	753.0	$\approx$ 64.4 ms	$\approx$ 5.42 s	$\approx$ 53.8 s

We report average per-constrain proving time and verification time of $1, 10^2$ and $10^3$ proofs for all zk-SNARKs with several elliptic curves. The benchmarks are done with an R1CS instance with 400.000 constrains and 10 input values, and the average of proving times are taken for 100 iterations and the verification for $10^3$ iterations. Proof generation is done in multi-thread setting with 16 threads, while the verification is done in the single-thread setting

*EC* Elliptic curve, *SE* Simulation extractability, *PCPT* Per-constraint proving time, *Ver.* Verifying, *ns* nanosecond, *ms* millisecond, *s* seconds, *B* Byte, *WSE* Weak simulation extractable, *SSE* Strong simulation extractable, *AGM* Algebraic group model, *GGM* Generic group model, *RO* Random oracle, *CRH* Collision resistant hash. Among the strong SE ones, we have highlighted the most efficient verification

We evaluate the efficiency of our presented simulation extractable variants of Groth’s zk-SNARK using a prototype implementation in $\textsf{Arkworks}$^3^ which is an ecosystem written in Rust for developing and programming with zk-SNARKs. A prototype implementation of both $\textsf{Groth16}$ [22] and Groth and Maller’s zk-SNARK [23] are already presented in $\textsf{Arkworks}$ library, and in order to obtain a fair comparison and a comprehensive outcome, we also present an efficient implementation of Bowe and Gabizon’s construction [14] and our initial construction [7] in the same library.^4^

Our empirical analysis are done with the elliptic curves BLS12-381, MNT4-298, MNT6-298, MNT4-753 and MNT6-753 that BLS12-381 is estimated to achieve between 117 and 120 bits security [30], and the other four curves are estimated to achieve respectively $2^{77}$, $2^{87}$, $2^{113}$, $2^{137}$ security [9]. All experiments are done on a desktop machine with Ubuntu 20.4.2 LTS, an Intel Core i9-9900 processor at base frequency 3.1 GHz, and 128GB of memory. Proof generations are done in the multi-thread mode, with 16 threads, while proof verifications are done in a single-thread mode.

Following the benchmark strategy in Arkworks library, we report *Per-Constraint Proving Time* (PCPT) and *verification time* for both the proposed constructions in Sects. 3 and Appendix A and compare their efficiency with (weak or strong) SE zk-SNARKs of $\textsf{Groth16}$ [22], Groth-Maller (GM17) [23] and Bowe-Gabizon (BG18) [14]. Motivated by blockchain and large-scale applications like Zcash [8], we also compare (deterministic) verifying time of all constructions for the case that one needs to verify a large number of proofs for a particular language simultaneously. In the verification step of our constructions, one needs to compute exponentiation in $\mathbb {G}_2$ and $\mathbb {G}_T$, which can be optimized by Multi-Scalar Multiplication (MSM) techniques.

Table 2 presents an empirical analysis of our constructions and compares them with several relevant SE zk-SNARKs for an R1CS instance with 400.000 constraints and 10 input variables. The reported times are the average values on 100 iterations for proof generation and 10.000 iterations for verification. As it can be seen, similar to BG18 construction [14], provers of our constructions are almost as efficient as Groth’s protocol, while due to a different NP characterization, the GM17 scheme is considerably less efficient in comparison with other schemes. For instance, to generate a proof for an arithmetic circuit with 400.000 constraints, with BLS12-381 curve, $\textsf{Groth16}$, BG18, and both of our constructions require $\approx ~2.01$ seconds, while GM17 needs $\approx ~4.41$ seconds.

Among the compared strong SE constructions, GM17 has the shortest proof size, namely 2 elements from $\mathbb {G}_1$ and 1 element from $\mathbb {G}_2$, and our construction in Sect. 3 has the second shortest proof size, namely 2 elements from $\mathbb {G}_1$ and 2 elements from $\mathbb {G}_2$.

In the last two columns of Tab. 2, we report the verification time of all constructions for the case that we need to verify $10^2$ or $10^3$ proofs of the same language. Once verifying a large number of proofs, our constructions use the MSM technique to compute the needed exponentiations in all proofs at the same time, which allows us to save on total verification time. As it can be seen, our construction presented in Sect. 3 has the most efficient verification among the strong SE constructions, and above all in the case of verifying a large number of proofs, the total verification time in both of our constructions improve significantly using the MSM technique. In particular, the verification of our second construction has very close efficiency to the original $\textsf{Groth16}$. For instance, in the case of BLS12-381, once we verify 100 proofs, the total verification time for $\textsf{Groth16}$ is $\approx 0.190$ seconds, and for our second construction is $\approx 0.194$. As it can be seen the gap is small and actually the larger the number of proofs we verify, the smaller this gap gets.

**Fig. 2:**
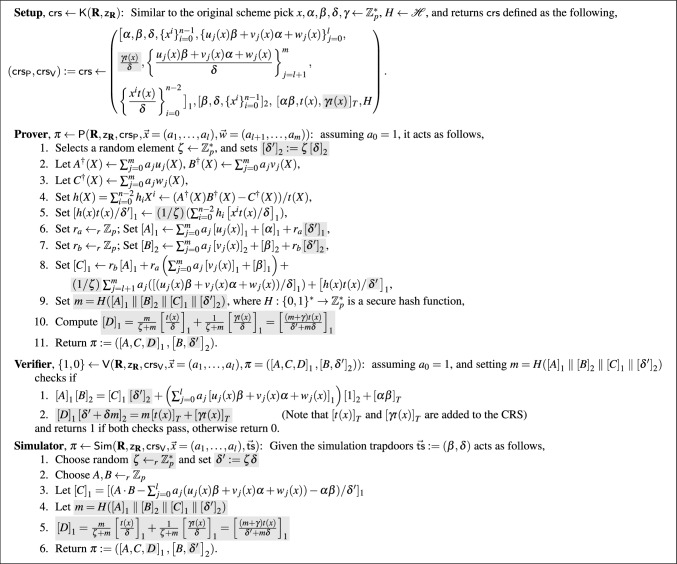
Our initial strong SE variant of $\textsf{Groth16}$ for $\textbf{R}$ along with a modification of the Boneh-Boyen signature. In the protocol, $\mathscr {H}$ is a family of collision resistant hash functions that map to $\mathbb {Z}_p^*$ [7]

## Conclusion

Over the last few years, various SE zk-SNARKs have been proposed that achieve (strong) simulation extractability [2, 14, 23, 29], which is a security property stronger than knowledge soundness and prevents attacks from the adversaries who have seen simulated proofs. Simulation extractability implies non-malleability of proofs [23] and its variant with *black-box extraction* is shown to be sufficient for achieving UC-security in NIZK arguments [21]. SE zk-SNARKs allow us to build succinct signature-of-knowledge schemes [16, 23], and they can also be used to build chameleon hash functions [25].

In this paper, we revised the SE variation of $\textsf{Groth16}$ proposed in [14] and presented a new variation. Our initial construction from CANS 2020 ( [7], Appendix A) requires 4 pairings in verification, instead of 5 in [14], and also avoids random oracles in exchange for using a collision resistant hash function. It has a more efficient prover, $\textsf{crs}$ size, and proof size in comparison with [2], that has also 4 pairings in the verification. Our new variant used some subtle modifications to shorten the proof size and improved the verification of Bowe and Gabizon’s construction significantly [14]. In this variant, we showed that using a random oracle, we can achieve strong SE in $\textsf{Groth16}$, at the cost of one additional $\mathbb {G}_2$ element in the proof, and one new exponentiation in $\mathbb {G}_2$ in the verification, where the later introduces negligible overhead to the verification of $\textsf{Groth16}$ in the cases that one needs to verify a large number of proofs for the same circuit (e.g. Zcash [8]).

We evaluated the empirical performance of our constructions in Arkworks library. Our evaluations showed that our constructions are among the most efficient SE zk-SNARKs. Particularly, in large-scale applications, the CRS, the prover, and the verifier of our new SE zk-SNARK are almost as efficient as the original $\textsf{Groth16}$. Just, in our case the proof consists of 4 group elements, instead of 3 in the original construction of $\textsf{Groth16}$. This seems to be a minimal cost to achieve *strong* SE in $\textsf{Groth16}$.

## Data Availability

All data generated or analyzed during this study are included in this paper and the source codes of implementations are publicly available on https://github.com/Baghery/ABPR22.

## Appendix A: A simulation extractable zk-SNARK without RO

In this section, we recall the construction of our first (strong) SE variant of $\textsf{Groth16}$ based on Bowe and Gabizon’s scheme [14] which is presented in [7].

***Scheme Definition***. In Fig. 2, we recall the construction of our first variation of $\textsf{Groth16}$ [7], that similarly works with quadratic arithmetic programs. In this construction, the Proof of Knowledge (PoK) of the DLOG of $[\delta ']_2$ w.r.t. $[\delta ]_2$ is changed to another PoK in the GGM that relies on the collision resistance property of the hash function. In Fig. 2, the elements $[\alpha \beta ,t(x), \gamma t(x)]_T$ are redundant and can in fact be computed from the rest of the elements in the $\textsf{crs}$. Alternatively, one can describe $\textsf{Groth16}$ as corresponding to $\zeta =1,\gamma =0$ and where the proof consists only of $\left[ {A}, {C}\right] _{1}, \left[ {B}\right] _{2}$. Differences with $\textsf{Groth16}$ are highlighted. We briefly give an intuition behind the construction in the following.

***Avoiding Random Oracle***. In [7], it is proven that the variation of $\textsf{Groth16}$ described in Fig. 2, guarantees (1) perfect completeness, (2) perfect zero-knowledge and (3) simulation-extractability in the asymmetric GGM. The proof of construction uses the collision resistance property of the hash function and the GGM. Roughly speaking, the new variable $\gamma$ gives some additional guarantees because to compute $t(x) \frac{(\gamma +m)}{(\delta '+\delta m)}$ from $D_j$ such that $m_j \ne m$, it is necessary to know both $\frac{1}{(\delta '+\delta m)}$ and $\frac{\gamma }{(\delta '+\delta m)}$, but this is only possible when $\delta '+\delta m=k \delta$. Then, either one has the knowledge of the DLOG of $\delta '$ respect to $\delta$ ($k-m$), which is straightforward, or either one has re-used $\delta '_j$ and $m_j$ from some *j*th query. The last case is discarded when one reaches that same message had to be re-used, $m=m_j$, which breaks collision resistance of the hash.

## Footnotes

1 In the worst case, our changes add only one element to the $\textsf{crs}$ of $\textsf{Groth16}$ and since $\textsf{Groth16}$ is already proven to achieve subversion ZK (ZK without trusting a third party) [1, 18], our variants also can be proven to achieve Sub-ZK using the technique proposed in [4, 18].

2 These new group elements can be seen as additional independent variables. Using the same trick as [19], the multivariate case can be reduced to the univariate one by writing each new variable as a degree one polynomial of the same variable. These additional variables ultimately do not change the total degree of the polynomial that the adversary constructs as his output, which is what determines the loss in the reduction.

3 Available on https://github.com/arkworks-rs.

4 Source codes of our implementations are publicly available on: https://github.com/Baghery/ABPR22.
